# LncRCD: A Comprehensive Database for Pan-Cancer Characterization of lncRNAs Related to 12 Regulated Cell Death Types

**DOI:** 10.34133/csbj.0110

**Published:** 2026-06-01

**Authors:** Hongying Zhao, Lin Bai, Shiyi Li, Yanwu Sun, Wangyang Liu, Zushun Chen, Lu Wang, Chuncheng Hao, Li Wang

**Affiliations:** ^1^ College of Bioinformatics Science and Technology, Harbin Medical University, Harbin 150081, China.; ^2^ Department of Head and Neck Radiation Oncology, Harbin Medical University Cancer Hospital, Harbin 150081, China.

## Abstract

Regulated cell death (RCD) is a fundamental biological process that determines tumor progression and treatment response. Although high-throughput sequencing technologies have revealed a large number of tumor-related long noncoding RNAs (lncRNAs), systematically analyzing the regulatory landscape of lncRNAs under multiple RCD patterns remains a challenge. Here, we present LncRCD (https://lncrcddb.bio-database.com/), a comprehensive resource and analysis platform specifically for cancer cell death-related lncRNAs. This study systematically integrated 1,595 core genes involved in 12 types of RCD and identified 4,624 pairs of RCD–lncRNA regulatory relationships in 18 cancer types, covering 2,088 lncRNAs with potential functional significance. To demonstrate the clinical translational value of this large-scale dataset, we conducted a comprehensive downstream bioinformatics analysis, including constructing a robust prognostic evaluation model based on RCD–lncRNA signatures, using the non-negative matrix factorization (NMF) algorithm to identify molecular subtypes with unique immune characteristics and survival outcomes, and predicting potential treatment drug sensitivity based on cancer treatment response portal data, thereby linking molecular phenotypes to clinical therapeutic guidance. The LncRCD database is a comprehensive resource database and a discovery-oriented platform, integrating user-friendly search, analysis, browsing, download, and visualization functions. Its aim is to provide a convenient resource for exploring the complex regulatory relationships between RCD and lncRNAs in human cancers. Ultimately, this study not only presents a panoramic view of lncRNAs participating in the regulation of multiple RCD patterns but also provides a valuable resource for linking omics data to biological interpretation, which may help elucidate tumor death mechanisms and offer insights for future precision immuno-oncology strategies.

## Introduction

Regulated cell death (RCD) is a form of cell death caused by the activation of one or more signal modules that can be pharmacologically or genetically regulated [1]. The induction and execution of RCD is mainly regulated by the formation of signal amplification complexes, which play an important role in development and immune response [2–4]. When mammalian cells are exposed to irreversible disturbances in the intracellular or extracellular microenvironment, one of many signal cascades can be activated and eventually lead to cell death [5,6]. Different types of RCD exhibit a full spectrum of morphological features ranging from complete necrosis to full apoptosis, as well as immunomodulatory features ranging from anti-inflammatory and tolerant to pro-inflammatory and immunogenic [7–10]. Due to the modifiable and targetable nature of RCD, more and more studies are focusing on the regulatory pathways and characteristics of RCD types in cancer [5,9,11–13]. Currently, many researchers have identified several therapeutic targets and pathways for RCD [14–16]. At the same time, new evidence indicates that there is a complex “cross-talk” between different death modes, such as ferroptosis, necroptosis, and pyroptosis [17–19], which cooperate in shaping the tumor immune microenvironment. Therefore, a panoramic “pan-RCD” perspective is crucial for deciphering the molecular logic of tumor degeneration and developing strategies to overcome multi-drug resistance by jointly triggering multiple RCD pathways.

Long noncoding RNA (LncRNAs) are a class of long-stranded noncoding RNAs greater than 200 nucleotides in length, which are important regulators of gene expression at different levels [20]. Abnormal expression and mutation of lncRNAs are closely related to tumorigenesis, metastasis, and tumor staging [21–25]. lncRNAs can modulate genes through diverse mechanisms, including functioning as competitive endogenous RNAs (ceRNAs) to sequester microRNAs [26–29], serving as molecular scaffolds for protein complexes [30–33], or inducing epigenetic modifications [34–36]. However, the mechanism of action and clinical application of RCD-related lncRNAs in cancer still need further in-depth research and exploration, with a view to providing more precise methods and strategies for early diagnosis, treatment, and prognosis assessment of cancer. The regulatory landscape of RCD is further complicated by the involvement of lncRNAs, which act as versatile conductors in gene expression [37–39]. For instance, specific lncRNAs have been shown to sensitize tumor cells to apoptosis or ferroptosis by stabilizing key transcription factors [40–42] or enzymes [43,44]. Despite these sporadic findings, a systematic characterization of the lncRNA-mediated regulatory network across multiple RCD patterns remains largely unexplored, leaving a significant gap in our understanding of noncoding regulatory layers in cell death.

**Fig. 1.**
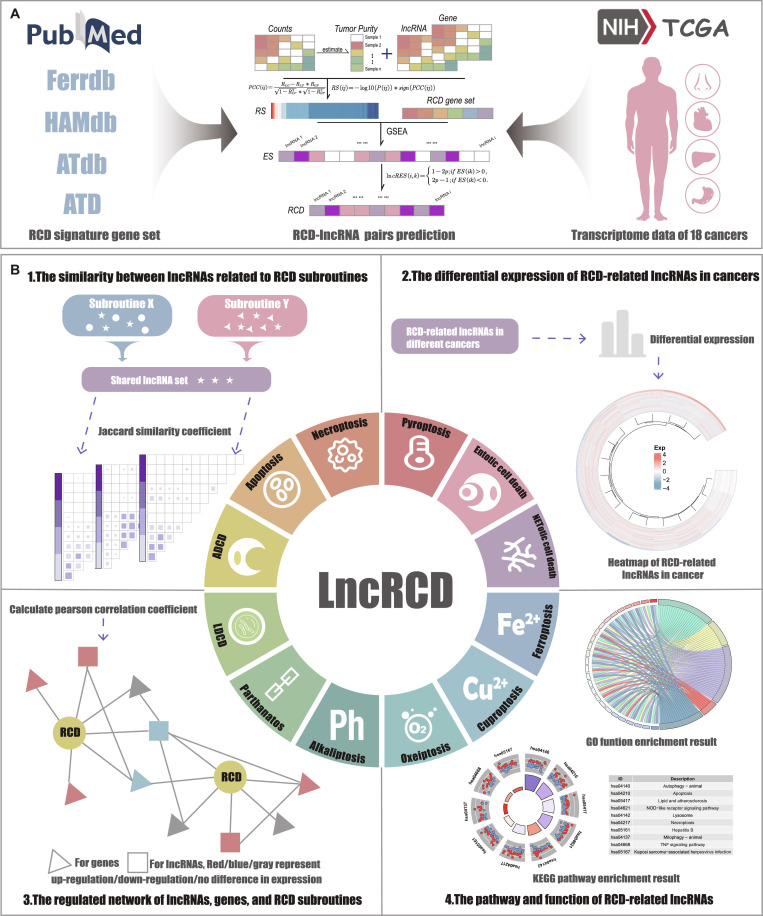
LncRCD analysis process and lncRNA spectra related to RCD in multiple cancer types. (A) The sources of 12 types of regulatory cell death and related gene sets are from PubMed and multiple databases. Predict RCD-related lncRNAs by combining RCD-related genes and TCGA data. (B) LncRCD also contains the similarity between lncRNA sets related to RCD types, the differential expression of lncRNAs related to RCD types, the regulated network of RCD types, RCD-related lncRNAs and RCD-related genes, and the functional annotations of lncRNAs related to RCD types.

While several databases have cataloged RCD-related protein-coding genes [45,46], a specialized platform dedicated to the RCD–lncRNA interface is still lacking. Existing resources often provide fragmented information, failing to offer interactive analysis tools for cross-cancer comparisons or clinical correlation studies. Therefore, there is an urgent need for a comprehensive resource that bridges the gap between high-throughput sequencing data and actionable biological insights. To address this issue, we have developed LncRCD, a manually curated resource of RCD-related genes and RCD-related lncRNAs in various cancers (Fig. 1). As summarized in Fig. 1, LncRCD integrates curated RCD gene sets and transcriptomic data from 18 cancers to identify RCD-related lncRNAs, and further provides similarity analysis across RCD types, differential expression patterns, regulatory networks, and functional enrichment results. This resource provides, for the first time, RCD-related genes and lncRNAs from literature and algorithmic predictions. LncRCD is designed as a systematic resource library and a discovery-oriented platform, with its main goal being to provide a standardized, pre-computed lncRNA–RCD interaction map, and may serve as a valuable tool for exploring the potential regulatory roles of lncRNAs in cancer, generating hypotheses on their involvement in cell death processes, and guiding the identification of putative biomarkers.

## Materials and Methods

### RCD-related gene sources

In order to build a comprehensive and authoritative RCD gene database, we adopted a multi-step, systematic approach, combining systematic literature mining with integration from professional public databases.

1. Systematic literature search and manual screening

We conducted a comprehensive search of PubMed (https://pubmed.ncbi.nlm.nih.gov/) using specific keywords for 12 RCD types: “regulated cell death” “RCD”. From 2,464 articles, we selected genes related to RCD that were either predicted or experimentally verified. Only genes that appeared in both independent studies would be included in the research.

2. Integration of professional RCD databases

The initial candidate list was cross-validated and integrated with genes from several mature RCD-related databases: FerrDb (version v1.0) [47]: for ferroptosis-related genes; Human Autophagy Modulator database (HAMdb) [46], Autophagy and Tumor Database (ATdb) [48], and Autophagy To Disease database (ATD) [49]: for autophagy-related genes.

3. Integration of RCD-related genes

We integrated the manually screened RCD genes with those provided in the RCD databases. When a gene appeared in different RCDs, the one-to-many relationship was retained, while duplicate cases where the gene and RCD were the same were deleted.

### Data acquisition and preprocessing

We obtained transcriptomic and clinical data from various cancer cohorts using the University of California, Santa Cruz (UCSC) XENA platform (https://xenabrowser.net/), with a particular focus on the Genomic Data Commons (GDC) The Cancer Genome Atlas (TCGA) dataset. To support differential expression analysis and correlation calculations, we downloaded the STAR-counts and STAR-FPKM expression profiles. In terms of gene annotation and transcript mapping, we used the GENCODE (v36) reference version, which provides a high-resolution catalog of lncRNA and mRNA. To ensure the integrity of the data and the uniqueness of downstream analysis mapping, if multiple transcripts or probes map to the same gene symbol, the average expression value is calculated to represent the unique level of the gene. Additionally, corresponding clinical metadata, including patient survival outcomes and phenotypic information, were integrated to facilitate subsequent case studies and clinical correlation verification. To ensure the reliability of our statistical analysis and to guarantee sufficient capacity to identify lncRNAs related to RCD, we established strict sample size screening criteria. Specifically, only cancer types that contain at least 5 samples in both the tumor group and the matched adjacent normal group were included in this study. This screening process ultimately enabled us to select 18 cancer types for comprehensive analysis.

### Identify RCD-related lncRNAs

We defined RCD-related lncRNAs as those whose expression levels show significant correlation with the expression of curated RCD-related genes across cancer samples. The rationale is that genes with coordinated expression patterns are often functionally related or co-regulated within the same biological processes or signaling pathways [50]. Therefore, such correlations provide a first-line strategy to infer potential functional involvement of lncRNAs in RCD.

First, we used DESeq2 [51] to analyze the differentially expressed genes between normal samples and tumor samples for count data of 18 types of cancers. We used adjusted *P* value < 0.05 and |log_2_FC| > 2 to screen out differentially expressed lncRNAs. The correction method of *P* value is Benjamini–Hochberg (BH). These high-frequency alterations of lncRNAs are more likely to play significant roles in biology.

Next, we used the ImmLnc [52] method to calculate the lncRNAs related to RCD (Supplementary Method). In particular, given the obvious heterogeneity of tumor samples themselves, we included tumor purity as a key covariate in some of the correlation analyses. We set the thresholds of |lncRES| > 0.995 and adjusted *P* value < 0.05 (BH) to ensure that the screened pairs of lncRNA–RCD types were significant. To empirically prove the rationality of the strict threshold used to define the significant lncRNA–RCD relationship, we conducted a sensitivity analysis. We tested different cutoff values of the |lncRES|, ranging from 0.990 to 0.999. The selected threshold was |lncRES| > 0.995 as an optimal empirical balance, strictly controlling the false discovery rate while retaining a robust core network of RCD–lncRNAs, suitable for downstream analysis (Fig. S1A).

### Multi-dimensional evidence integration and confidence stratification of RCD-related lncRNAs

To further screen the RCD-related lncRNAs with biological functions and reduce the risk of false positives caused by computational predictions, we constructed an evaluation system that integrates external experimental evidence and genomic spatial location information. This system conducts in-depth assessment of candidate lncRNAs from the following 3 dimensions.

1. Disease and cancer association annotation

We evaluated the known functions of candidate lncRNAs in human diseases (particularly cancer) by searching 3 authoritative manually verified databases: EVLncRNAs (v3.0) [53], Lnc2cancer (v3.0) [54], and LncRNADisease (v3.0) [55]. Only lncRNAs recorded in at least one of these databases were considered to have potential pathophysiological significance and were assigned corresponding confidence labels.

2. Experimentally validated regulatory target identification

To elevate the statistical correlation to functional interaction, we validated the RCD-related lncRNA–mRNA relationships using the LncTard (v2.0) [28] database. lncTard is a database specifically collecting experimentally verified lncRNA–target regulatory relationships (including interference, overexpression, and luciferase reporter gene detection). We cross-compared the lncRNA–RCD gene pairs identified by partial correlation analysis with this database. If a pair of related relationships has experimental evidence support in the database, it is defined as an experimentally validated RCD-related lncRNA–mRNA relationship pair.

3. Genomic proximity and cis-regulation analysis

Based on the biological characteristic that lncRNAs often exert cis-regulatory effects on neighboring genes, we calculated the physical distance between candidate lncRNAs and RCD genes on the chromosome. We downloaded the human genome annotation GTF file from the GENCODE (v38) database and extracted the start and end coordinates of each transcript using the GenomicRanges software package in R. Spatial classification criteria: According to the physical distance (Distance), we classified the related pairs into 4 categories:

Overlap/cis: lncRNA and RCD gene have overlap in genomic coordinates;

Cis_Proximal: lncRNA is upstream or downstream of RCD gene within a 100-kb range;

Cis_Distal: distance is between 100 and 500 kb;

Trans: distance exceeds 500 kb or on different chromosomes.

The proximity of physical distance (especially <100 kb) is regarded as strong positive evidence supporting the results of the correlation analysis, indicating that this lncRNA may directly regulate the expression of neighboring RCD genes through chromatin remodeling or recruitment of transcription factors. The Trans label indicates that the lncRNA may regulate the target RCD gene through a distant trans-regulatory mode.

Based on the above multi-dimensional evidence, we classified the RCD-related lncRNAs into 3 levels:

High confidence: Selected through correlation analysis and meeting all 3 dimensions simultaneously;

Medium confidence: Selected through correlation analysis and meeting at least 2 of the above dimensions;

Low confidence: Selected through correlation analysis and meeting any one of the above dimensions;

Candidate: Selected only through correlation analysis.

### Calculate the correlation coefficient between lncRNA and genes

We calculated the correlation coefficients between RCD-related genes and RCD-related lncRNAs to construct a regulatory network. We extracted the expression profiles of 1,595 genes and RCD-related lncRNAs in each cancer. Then, Pearson correlation coefficients (PCCs) were used to calculate the correlation coefficient between RCD-related genes and RCD-related lncRNAs [56]. Pearson correlation was adopted for its interpretability, robustness, and computational efficiency and is widely used in large-scale transcriptomic analyses to identify coordinated gene expression patterns and biologically meaningful modules [56–58]. It is considered a standard approach for gene coexpression analysis and has demonstrated stable performance in bulk RNA-seq datasets [59,60]. To empirically prove the rationality of the threshold used to define the correlation relationship between significant lncRNA–mRNA, we conducted a sensitivity analysis.

To empirically prove the rationality of the threshold used to define the correlation relationship between significant lncRNA–mRNA, we conducted a sensitivity analysis. We tested different cutoff values for the absolute correlation values, ranging from 0.4 to 0.7. The selected threshold was |PCC | > 0.4, which served as an optimal empirical balance value. It strictly controlled the false discovery rate while preserving the robust core network of lncRNA–mRNA and was suitable for downstream enrichment analysis (Fig. S1B). Finally, we set the threshold of correlated networks with |PCC| > 0.4 and adjusted *P* value < 0.05 (BH) as the criterion for the correlation between RCD-related genes and lncRNAs.

### Enrichment analysis

We used the clusterProfiler package for Gene Ontology (GO) and Kyoto Encyclopedia of Genes and Genomes (KEGG) pathway enrichment analyses [61–63]. We first collated the RCD-related genes that appeared in the regulatory network. The enrichment is enriched using clusterProfiler. The threshold of both *P* value and adjusted *P* value(BH) was set at <0.05. After obtaining the GO and KEGG pathway enrichment results of the regulatory networks of 18 cancers, we use the fgsea package to perform Gene Set Enrichment Analysis (GSEA) analysis. Tumor and normal samples from each cancer type were defined as the 2 phenotypes. The lncRNA expression matrix was used as input, and lncRNAs were ranked according to their differential expression fold changes between tumor and normal samples. Then, the enrichment scores of gene sets related to each RCD type were calculated based on this ranked list to evaluate the differential activity. RCD types showing significant differences in cancer were identified using a threshold of *P* < 0.05.

### Calculate Jaccard correlations between RCD types

We calculated the Jaccard similarity coefficient of lncRNA related to RCD type in each cancer to explore the overlap of lncRNAs among types. Given 2 RCD-related lncRNA sets A and B, Jaccard similarity coefficient is defined as the ratio of the intersection size of A and B to the union size of A and B, as follows:

$$J\;\left(A,\;B\right)=\;\frac{\left|A\cap B\right|}{\left|A\cup B\right|}=\;\frac{\left|A\cap B\right|}{\left|A|+|B|-|A\cap B\right|}$$ (1)

### Construct prognostic model

We summarized the lncRNAs related to differentially expressed RCD types and removed the duplicated lncRNAs. The tumor samples were randomly divided into a training set and a testing set at a ratio of 1:1. In the training set, univariate Cox regression analysis was performed using the survival package to identify RCD-related lncRNAs significantly associated with prognosis (*P* < 0.05). These candidate prognostic lncRNAs were further screened by least absolute shrinkage and selection operator (LASSO) Cox regression analysis using the glmnet package. Subsequently, the selected lncRNAs were incorporated into a multivariate Cox regression model to construct the prognostic signature. The regression coefficients derived from the multivariate Cox regression model were used to calculate the risk score for each patient. The risk score of this prognostic model is calculated as follows:

$$\text{risk}\_\text{score}=\sum \beta_{i}*X_{i}$$ (2)

where *β*_*i*_ represents the multiple Cox regression beta value corresponding to lncRNA. *X*_*i*_ indicates the expression value of lncRNA. The optimal cutoff value of the risk score was determined in the training set using the survminer package based on survival outcomes, and patients were subsequently classified into high-risk and low-risk groups.

### Non-negative matrix factorization

We used the prognosis-related lncRNAs (*P* < 0.05) for unsupervised molecular subtyping with the NMF package [64]. For NMF, we set the parameters as follows: Select the default “brunet” algorithm, 150 runs per rank, and set the number of clusters *K* to 2 to 8. The optimal rank was selected based on the cophenetic correlation coefficient, in combination with dispersion, silhouette, residual sum of squares (RSS), sparseness, and the consensus matrix, and *k* = 3 was chosen for downstream analysis. Samples were assigned to subtypes according to the final NMF solution, and representative subtype-defining lncRNAs were identified from the basis matrix based on their contribution to the clustering structure.

Clustering stability was further assessed by repeating NMF with multiple nrun (10, 30, 50, 100, 150, 200, 300) and calculating the adjusted Rand index (ARI) across runs. Subsampling-based robustness analysis was additionally performed, and the NMF results were compared with those from hierarchical clustering and *K*-means clustering, confirming the reproducibility and robustness of the 3-subtype classification.

### Prediction of drugs targeting lncRNA

In order to further explore the relationship between RCD-related lncRNAs and drugs, we chose the oncoPredict [65] method for calculation. We obtained drug response matrix as training data for the Cancer Therapeutics Response Portal (CTRP2) [66] from the website of oncoPredict. The CTRP2 dataset contains 51,847 genes, 829 cell lines, and 545 drugs, and these genes cover both coding and noncoding genes.

We used Wilcoxon rank-sum test and determined that those drugs with |log_2_FC| > 0.25 and *P* < 0.05 showed differences in sensitivity in cancer due to different levels of expression of RCD-related lncRNAs. Specifically, samples were divided into high-expression and low-expression groups according to the median expression value of each RCD-related lncRNA. The predicted drug response between the 2 groups was then compared, and log_2_FC was calculated as the log_2_-transformed ratio of the mean predicted drug response in the high-expression group to that in the low-expression group.

In addition to identifying drugs that show differences in sensitivity due to different expression values of RCD-related lncRNAs, we also calculated the correlation between RCD-related lncRNAs and drugs. Spearman correlation coefficient (SCC) was used as a measure of correlation. For those with SCC < −0.3 and *P* < 0.05, we consider them as candidate drugs potentially related to cancer treatment. Finally, drug–lncRNA pairs meeting both the differential sensitivity criteria (|log_2_FC| > 0.25 and *P* < 0.05) and the correlation criteria (SCC < −0.3 and *P* < 0.05) were retained as significant candidate associations.

### Database implementation

LncRCD is written using programming languages such as HyperText Markup Language (HTML), Cascading Style Sheets (CSS), and the scripting language JavaScript. Responsive data binding was implemented using Vue.js, and data were stored in MongoDB. By using Node.js and frameworks such as Express and Axios, the interaction between the front-end page and the MongoDB database has been achieved, enabling access and manipulation of data in the database. HTML is used to create web page structures, CSS is used to add styles to HTML documents, JavaScript is used to control the behavior of web pages, Vue.js is used to build user interfaces and single-page applications, and Node.js is used for the interaction between the front-end file system and the back-end database to achieve data storage and retrieval. MongoDB is a nonrelational database that uses distributed data storage and is one of the preferred databases for modern web applications. All the code is developed using VSCode (https://code.visualstudio.com/). In order to provide stable Web services, LncRCD is hosted on the CentOS-7 (https://www.centos.org/) operating system and uses services of versions such as Apache 2.4.53 (https://apache.org/), Node.js 2.6 (https://nodejs.org/en), and MongoDB 4.4.6 (https://www.mongodb.com/) to achieve database management and operation.

## Database Content and Usage

### Data structure and organization

The data of LncRCD mainly fall into 3 categories, namely, cancer, RCD, and lncRNA. A total of 1,595 genes related to 12 RCD types (apoptosis, necroptosis, pyroptosis, ferroptosis, cuproptosis, parthanatos, entotic cell death, NETotic cell death, lysosomal-dependent cell death, autophagy, alkaliptosis, and oxeiptosis) were included (Table S1). A total of 4,624 RCD type–lncRNA association pairs were identified across 18 TCGA cancer types, including bladder urothelial carcinoma, breast invasive carcinoma, cholangiocarcinoma, colon adenocarcinoma, esophageal carcinoma, glioblastoma multiforme, head and neck squamous cell carcinoma, kidney chromophobe, kidney renal clear cell carcinoma, kidney renal papillary cell carcinoma, liver hepatocellular carcinoma, lung adenocarcinoma, lung squamous cell carcinoma, prostate adenocarcinoma, rectum adenocarcinoma, stomach adenocarcinoma, thyroid carcinoma, and uterine corpus endometrial carcinoma (Fig. S1C and D). These RCD types–lncRNA relationships include 2,088 lncRNAs. We assigned corresponding evidence level labels to each identified lncRNA: “High confidence”, “Medium confidence”, “Low confidence”, or “Candidate”. In addition, the regulatory network contains the RCD type, RCD-related lncRNAs, and RCD-related genes. Among them, the relational pair data are stored in the MongoDB database using the String type, and the regulatory network and enrichment analysis data are directly displayed in a visual form on the page.

### Data archive and statistics

**Fig. 2.**
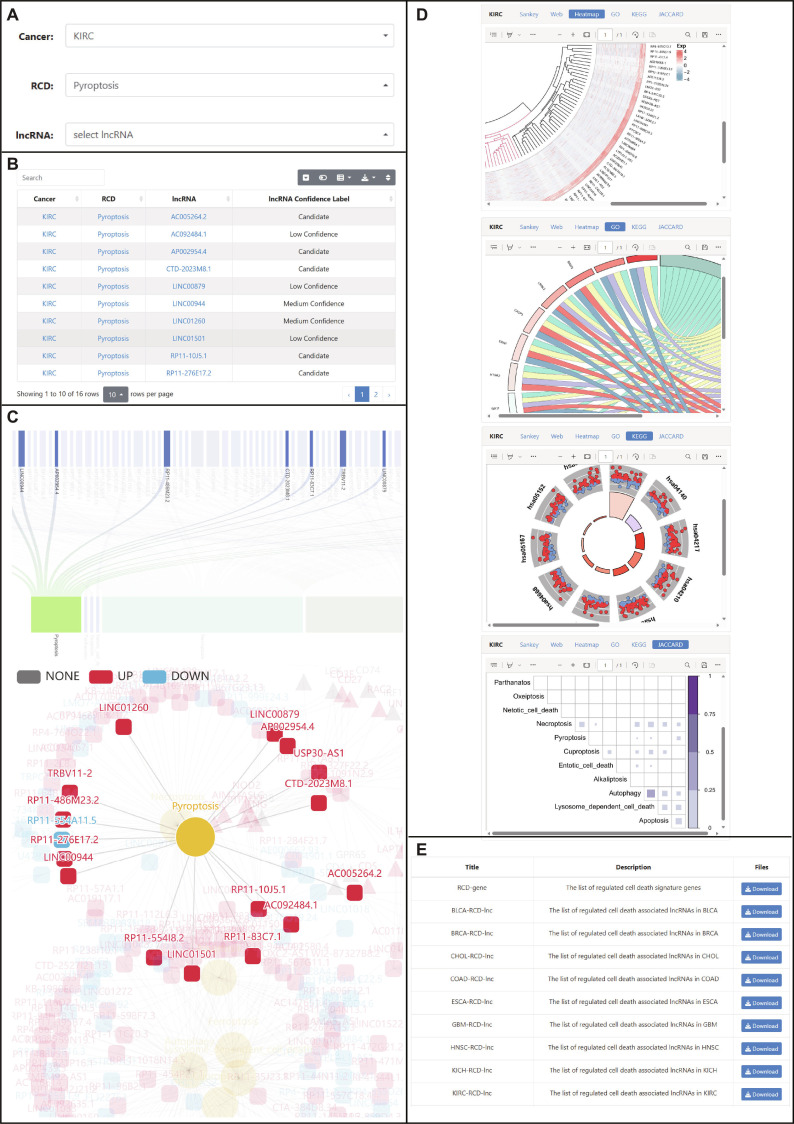
The workflow of LncRCD. (A) Users can search for specific relationship pairs between cancer, RCD, and lncRNAs. (B) The search results are presented in an interactive table, allowing for sorting and filtering. (C) Relevant cancer types and their associations can be explored through visual charts. (D) The relationships are detailed in an interactive Sankey diagram and a network graph, enabling users to manipulate the view and explore connections. (E) All the data are available for download on a dedicated page.

Users can directly use the LncRCD system without registration. LncRCD features a user-friendly interactive interface, allowing users to access, query, and analyze data conveniently: (a) On the search page, users can search for relationship pairs by selecting cancer, RCD, and lncRNA (Fig. 2A); (b) the search results are output in the form of a table (Fig. 2B); (c) after users click on the content of the table, they will be automatically redirected to the corresponding browsing page. On the browsing page, users can obtain visual charts related to cancer, including Sankey diagrams, network diagrams, and heat maps. The pictures support interactive operations. Users can explore the data by clicking and dragging with the mouse and can also download the pictures for further analysis (Fig. 2C and D). (d) On the download page, users can access all the data in the database at once or download them separately. The data include lncRNAs and genes related to RCD (Fig. 2E).

### Data submission and retrieval

As described above, users enter the search page, select “KIRC” from the cancer panel, choose “Pyroptosis” from the RCD panel, and leave the lncRNA panel blank to retrieve all relevant relationship pairs (Fig. 2A). After clicking the submit button, the system returns a results table listing all pyroptosis-related lncRNAs in KIRC (Fig. 2B). The download button above the table supports exporting the current query results in CSV format for local analysis. From the results table, users click on “Pyroptosis” in the RCD column, and the system automatically navigates to a dedicated visualization page. The sankey diagram illustrates the flow of associations among cancer types, RCD types, and lncRNAs, where users can click on any node to highlight related connections. The network graph displays the regulatory relationships between RCD-related genes and lncRNAs, supporting interactive operations such as dragging and zooming to facilitate exploration of complex regulatory networks. Additionally, this page provides enrichment analysis results for RCD-related genes in KIRC, with a download button above each chart supporting high-resolution image export. For users requiring comprehensive data access, the “Download” page integrates all datasets, including RCD-related lncRNAs, RCD-related genes, and regulatory relationship pairs across all 18 cancer types, packaged into a single compressed file (ZIP format) to facilitate large-scale batch analysis. The performance evaluation results of the LncRCD platform show that core functions respond quickly, with simple search completing in approximately 0.3 s, visualization page loading within 2.5 s, full dataset download in 0.5 s, and an average response time of only 36 ms under 10 concurrent users, meeting the demands of routine scientific research use.

Additionally, users can use command-line tools for automated downloading: curl -o LncRCD_data.zip https://lncrcddb.bio-database.com/download/all_data.zip. The downloaded CSV files can be directly imported into programming environments such as R or Python for custom analysis. Due to the extremely small data volume, a single download provides access to the complete dataset without requiring multiple API calls, fully meeting the batch analysis needs of computational researchers.

### Case study

**Fig. 3.**
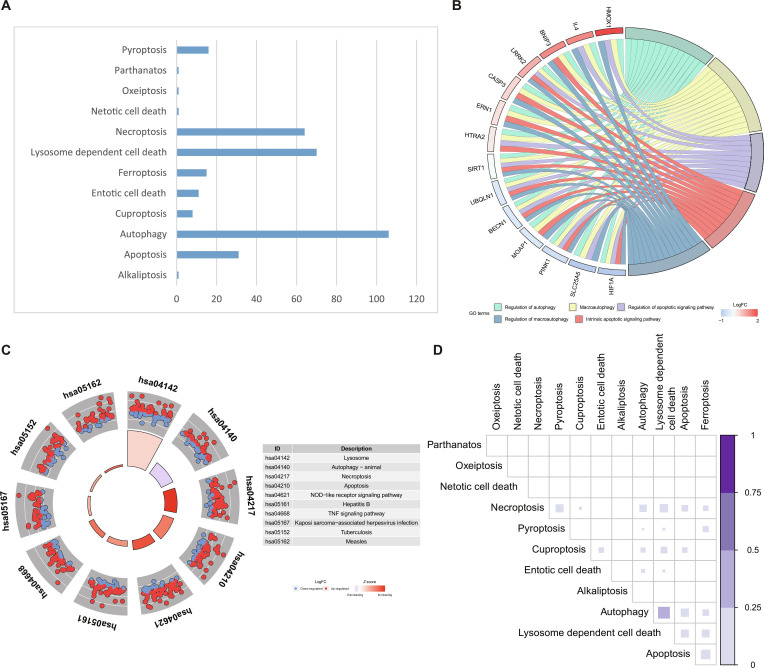
GO and KEGG enrichment results and overlap patterns among different RCD types in KIRC. (A) Number of RCD-related lncRNAs identified for each RCD type in KIRC. Autophagy contained the largest number of associated lncRNAs, followed by lysosome-dependent cell death and necroptosis. (B) Chord diagram illustrating the relationships between representative RCD-related genes and enriched GO biological process terms. (C) KEGG enrichment overview of RCD-related genes, showing significantly enriched pathways and the expression patterns of pathway-involved genes. (D) Pairwise overlap of RCD-related lncRNA sets among different RCD programs in KIRC. The size and color intensity of each square represent the degree of overlap between 2 corresponding lncRNA sets, with darker color indicating greater overlap.

As a typical example, KIRC demonstrates a clear and organized distribution pattern for lncRNAs related to RCD. We identified 325 pairs of RCD types and lncRNA correlations in KIRC, including 12 RCDs and 210 lncRNAs (Fig. 3A). We conducted GO and KEGG enrichment analyses on the genes in the constructed lncRNA–gene network to evaluate the potential biological functions of the RCD-related lncRNAs. The GO analysis indicated that these genes were mainly enriched in processes related to autophagy and apoptosis, including autophagy regulation, macroautophagy regulation, macroautophagy, regulation of apoptotic signaling pathways, and regulation of intrinsic apoptotic signaling pathways (Fig. 3B). The KEGG enrichment analysis revealed significant enrichment for pathways such as autophagy (animals), necroptosis, apoptosis, and the nucleotide-binding oligomerization domain (NOD)-like receptor signaling pathways (Fig. 3C). Several pathways related to infection were also identified, suggesting potential links between RCD-related genes and immune or stress response mechanisms. Additionally, we found that the identified genes might be involved in multiple interrelated biological processes of cell death rather than functioning independently in a single RCD category. Inspired by this phenomenon, we used Jarcard analysis and found overlapping situations of lncRNAs in different RCDs (Fig. 3D). For instance, in the pairs of RCDs such as necroptosis and pyroptosis, autophagy, and lysosome-dependent cell death, the lncRNAs were significantly shared, while other programs showed relatively less overlap. This pattern indicates that in KIRC, some lncRNAs may be involved in the coordinated regulation of multiple death pathways. The overlap between necroptosis and pyroptosis is biologically plausible because both pathways are closely associated with innate immune and inflammatory signaling. Pyroptosis is typically triggered by inflammasome activation in response to pathogen-associated molecular patterns (PAMPs) and damage-associated molecular patterns (DAMPs) [67], whereas necroptosis can be induced by inflammatory receptors and pattern-recognition pathways, including Toll-like receptor (TLR)-mediated signaling and infection-related stimuli [68]. Similarly, the close association between autophagy and lysosome-dependent cell death is consistent with their shared reliance on intracellular degradative processes and lysosomal activity [69,70]. These results collectively indicate that in KIRC, the RCD–lncRNA network not only demonstrates functional interconnections but also exhibits pathway specificity. This provides support for using KIRC as a useful case for downstream prognostic and therapeutic analysis.

**Fig. 4.**
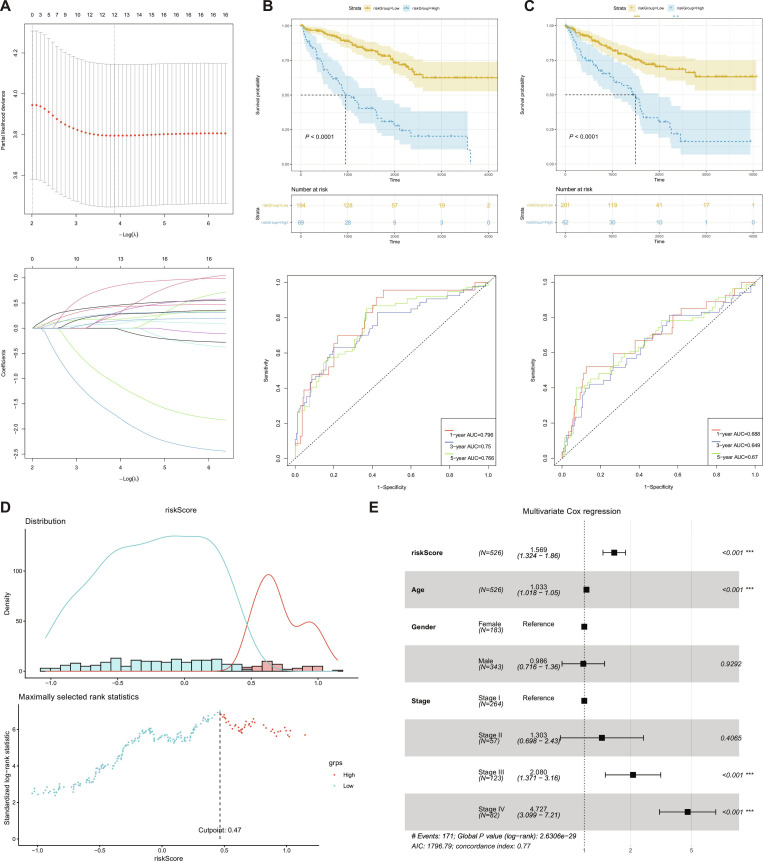
Construction, validation, and prognostic evaluation of the risk model. (A) Prognostic genes were selected using LASSO–Cox regression analysis. The upper panel shows the cross-validation curve for selecting the optimal penalty parameter λ, and the lower panel shows the coefficient profiles of candidate genes under different λ values. (B) Determination of the optimal cutoff value based on the risk score. The upper panel shows the distribution of risk scores in different risk groups, and the lower panel shows the maximally selected rank statistics used to identify the optimal cutoff. The dashed line indicates the optimal cutoff value. (C) Kaplan–Meier survival analysis and time-dependent ROC curves in the training cohort. Patients were stratified into high- and low-risk groups according to the optimal cutoff value, and the high-risk group showed significantly worse overall survival than the low-risk group. The lower panel presents the predictive performance of the model at 1, 3, and 5 years. (D) Kaplan–Meier survival analysis and time-dependent ROC curves in the validation cohort, demonstrating the robustness and predictive value of the risk model. (E) Forest plot of multivariate Cox regression analysis. After adjustment for age, sex, and clinical stage, the risk score remained an independent prognostic factor.

In KIRC, 4 RCD types were differentially expressed (*P* < 0.05): Apoptosis [normalized enrichment score (NES) = 1.258237], necroptosis (1.457494), and pyroptosis (1.553993) were up-regulated, while cuproptosis (−1.420964) was down-regulated by GSEA [71]. After removing duplicated RCD-related lncRNAs, expression profiles and survival data from 526 tumor samples were integrated for prognostic analysis. The samples were randomly divided into a training cohort and a testing cohort at a ratio of 1:1. Univariate Cox regression analysis was first performed to identify prognostic-related lncRNAs, followed by LASSO Cox regression analysis for further variable selection (Fig. 4A). Multivariate Cox regression analysis ultimately identified 9 prognostic lncRNAs, including RP11-417E7.1, RP11-291B21.2, CTD-2023M8.1, LINC00944, LINC01260, AC092484.1, RP11-867G23.13, RP11-1018N14.5, RP11-276E17.2, RP11-267A15.3, RP11-480D4.6, and LAMA5-AS1, which were used to construct the prognostic signature (*P* < 0.05; Table S2). Based on the optimal cutoff value of the risk score (cutoff = 0.4651601), patients were stratified into high- and low-risk groups (Fig. 4B). Kaplan–Meier survival analysis showed that patients in the high-risk group had significantly worse overall survival than those in the low-risk group in both the training and testing cohorts (both *P* < 0.0001; Fig. 4C and D). Time-dependent receiver operating characteristic (ROC) analysis demonstrated that the prognostic model had good predictive performance. In the training cohort, the area under the curve (AUC) values for 1-, 3-, and 5-year overall survival were 0.796, 0.750, and 0.706, respectively; in the testing cohort, the corresponding AUC values were 0.688, 0.649, and 0.670, respectively (Fig. 4C and D). Furthermore, multivariate Cox regression analysis showed that the risk score remained an independent prognostic factor after adjustment for clinical variables [hazard ratio (HR) = 1.553, 95% confidence interval (CI): 1.310 to 1.841, *P* = 3.97e−07; Fig. 4E].

**Fig. 5.**
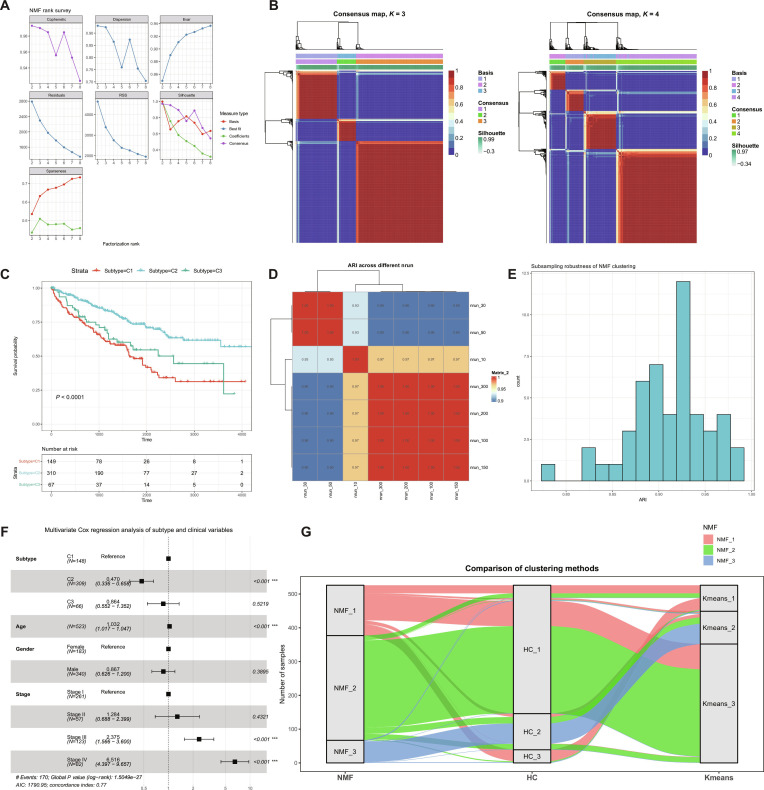
Identification and validation of molecular subtypes by NMF clustering. (A) Rank survey for NMF clustering. Multiple metrics were evaluated across different factorization ranks to determine the optimal number of clusters. (B) Consensus heatmaps generated by NMF clustering at *K* = 3 and *K* = 4. Each heatmap shows that compared with *K* = 4, the clustering structure at *K* = 3 displayed clearer block patterns and better stability. (C) Kaplan–Meier survival analysis of the 3 molecular subtypes. Significant differences in overall survival were observed among the 3 subtypes (*P* < 0.0001). (D) Heatmap of adjusted Rand index (ARI) values across different nrun. Pairwise ARI values were calculated to assess the reproducibility of NMF clustering under different initializations. (E) Distribution of pairwise ARI values across different NMF runs, showing generally high concordance among clustering results and supporting the robustness and stability of the 3-subtype solution. (F) Forest plot of multivariate Cox regression analysis. After adjustment for age, sex, and clinical stage, the subtype remained an independent prognostic factor. (G) Sankey diagram showing the concordance among sample assignments derived from NMF, hierarchical clustering (HC), and *K*-means clustering.

**Fig. 6.**
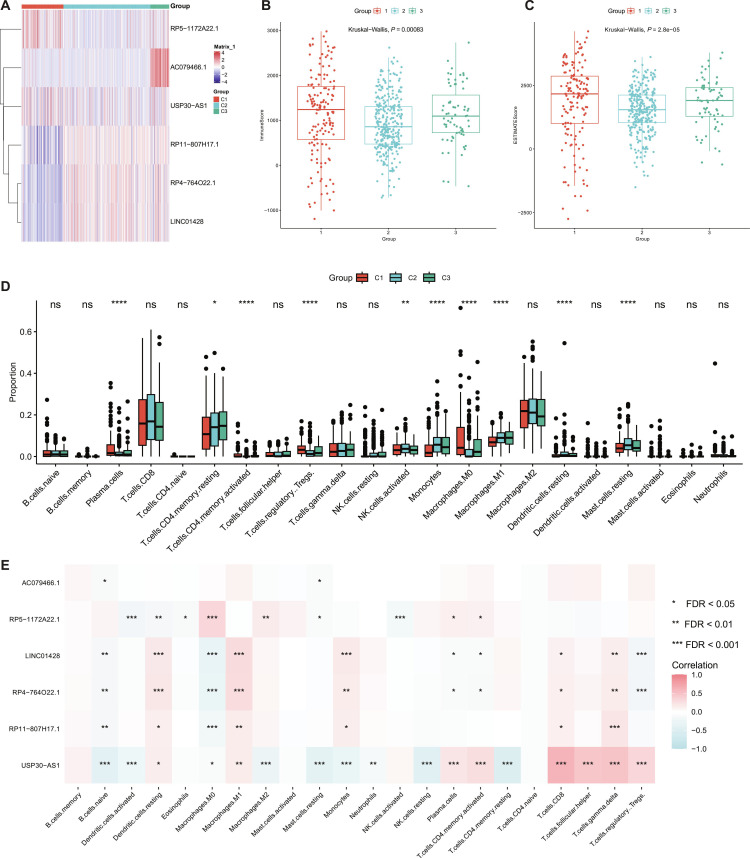
RCD-related lncRNAs classify molecular subtypes of cancer and the immune infiltration characteristics of KIRC subtypes. (A) Heatmap showing the expression of 6 characteristic lncRNAs across the 3 subtypes (C1, C2, and C3). The Immune Score (B) and ESTIMATE Score (C) of KIRC subtype samples. Statistical significance was determined by Kruskal–Wallis test. (D) The Differences composition in 22 types of immune cell of KIRC subtype. ****P < 0.0001, ***P < 0.001, **P < 0.01, *P < 0.05, ns not significant. (E) Correlation between lncRNA and 22 types of immune cell infiltration fraction of KIRC subtype. Spearman rank correlation coefficients were computed, and *P* values were adjusted for multiple testing using the BH procedure (FDR). ****FDR < 0.0001, ***FDR < 0.001, **FDR < 0.01, *FDR < 0.05, ns not significant.

In parallel, we applied NMF to 38 prognosis-associated RCD–lncRNAs across 526 KIRC tumors and defined 3 subtypes (Fig. 5A and B) driven by 6 lncRNAs (RP5-1172A22.1, RP11-807H17.1, RP4-764O22.1, LINC01428, USP30-AS1, and AC079466.1). Subtypes c1, c2, and c3 (*n* = 149, 310, and 67) showed significantly different overall survival (*P* < 0.0001; Fig. 5C). The 3-subtype solution showed good robustness across different nrun, with generally high pairwise ARI values (Fig. 5D and E), supporting the stability of the NMF clustering. To further assess the clinical relevance of the identified subtypes, we performed multivariate Cox regression analysis including subtype, age, gender, and pathological stage (Fig. 5F). Using C1 as the reference, C2 was independently associated with improved overall survival (HR = 0.470, 95% CI: 0.336 to 0.658, *P* < 0.001), whereas C3 showed no significant difference (HR = 0.864, 95% CI: 0.552 to 1.352, *P* = 0.5219). In addition, age and advanced pathological stage remained significant predictors of poor prognosis, while gender was not significantly associated with survival. These findings suggest that the identified subtype classification provides prognostic information beyond conventional clinicopathological characteristics. Comparison with hierarchical clustering and *K*-means further demonstrated a high degree of concordance in sample assignment, especially for the major subtype structure (Fig. 5G), indicating that the 3-cluster classification was reproducible across methods. The signature gene expressions and immune microenvironments of subtypes also differed (S6Fig. 6A to C and Table S3). Consistently, 18 of the 22 CIBERSORT cell types showed differences among the 3 subtypes (Fig. 6D and Table S4). Correlation analysis further indicated that these 6 lncRNAs defining the subtypes were widely correlated with the immune infiltration patterns (Fig. 6E). The 3 KIRC subtypes also exhibited distinct immune states. Subtype c1 was characterized by the highest ImmuneScore and ESTIMATEScore, with relatively increased plasma cells, resting CD4 memory T cells, and macrophage M0 infiltration, together with preferential expression of RP5-1172A22.1 and USP30-AS1, suggesting an inflamed but myeloid-remodeled state. In contrast, c2 showed the lowest immune and stromal scores and generally reduced activated immune cell infiltration, and was relatively associated with RP11-807H17.1, RP4-764O22.1, and LINC01428, consistent with a relatively immune-quiescent phenotype. By comparison, c3 displayed higher CD8^+^ T cells, activated CD4 memory T cells, activated natural killer (NK) cells, monocytes, M1 macrophages, and regulatory T cells (Tregs), together with prominent AC079466.1 expression, indicating an immune-active but concurrently regulated state. These immune-related patterns are biologically plausible, as immunogenic RCD programs, particularly pyroptosis and necroptosis, can promote inflammatory cytokine release, DAMP exposure, dendritic cell activation, and effector immune cell recruitment, while persistent inflammation may also engage compensatory immunoregulatory programs [9,17]. This interpretation is also consistent with the highly infiltrated and immunologically heterogeneous microenvironment previously reported in clear cell renal cell carcinoma (ccRCC) [72]. Meanwhile, the KIRC subtypes were overall associated with the mmc2 [73] immune subtypes but the concordance was low (Cramér’s *V* = 0.236; ARI = 0.102), suggesting that the 2 classification systems are related but not equivalent.

**Fig. 7.**
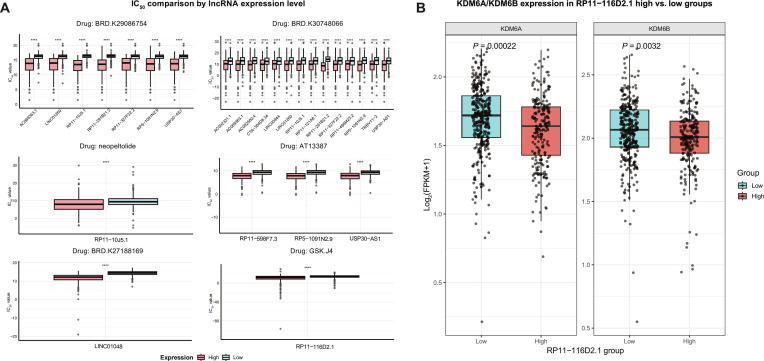
Predicted drug sensitivity and differential expression of epigenetic regulators associated with RP11-116D2.1. (A) Based on oncoPredict, expression-defined groups of RCD-related lncRNAs were associated with differences in estimated IC_50_ values for several candidate compounds. The plots show the predicted IC_50_ distributions for BRD-K2986754, BRD-K30748066, neopeltolide, AT13387, BRD-K27181869, and GSK-J4 across the corresponding high- and low-expression groups. Lower IC_50_ values in the high-expression group suggest increased predicted sensitivity under that transcriptional state. Statistical significance was determined by Wilcoxon rank-sum test. ****P < 0.0001, ***P < 0.001, **P < 0.01, *P < 0.05, ns not significant. (B) KDM6A and KDM6B expression differed significantly between the RP11-116D2.1 high- and low-expression groups. Jittered points overlaid on boxplots represent individual samples and overall distribution, and *P* values were derived from the Wilcoxon rank-sum test.

Finally, based on the oncoPredict method [65], we integrated RCD–lncRNA expression with drug sensitivity data in KIRC and, in KIRC, predicted 17 RCD-related lncRNAs–6 candidate drug association pairs (Table S5). Samples with higher lncRNA expression showed lower IC_50_ (median inhibitory concentration) values (Fig. 7A), suggesting that tumors with high RCD-related lncRNA expression may be more sensitive to the predicted agents. These results indicate that RCD–lncRNA profiles may help identify potential therapeutics for KIRC; however, therapeutic efficacy remains to be validated. Taking the predicted GSK-J4-RP11-116D2.1 relationship as an example, this prediction is biologically reasonable. Previous studies have shown that GSK-J4 can inhibit H3K27 dimethylase KDM6A/UTX and KDM6B/JMJD3, which are enzymes involved in the regulation of transcriptional programs related to inflammation and stress responses [74–76]. In our case study, the high expression group of RP11-116D2.1 had a lower predicted IC_50_ value for GSK-J4, decreased expression of KDM6A and KDM6B (Fig. 7B), increased enrichment of reactive oxygen species (ROS) and interleukin-6 (IL-6)/Janus kinase (JAK)/signal transducer and activator of transcription 3 (STAT3) signaling pathways, and relative inhibition of DNA repair (Table S6). These observations provide a biological background for linking our predictions to the known regulatory axis centered on KDM6A/B, but further experimental verification is needed to determine the underlying causal mechanism. Overall, our prediction results may help to prioritize the selection of candidate therapeutic drugs for KIRC. Further experiments and clinical validations are essential to confirm their actual therapeutic effects.

## Discussion

In this study, we systematically identified 2,088 lncRNAs associated with 12 types of RCD across 18 cancers using bulk transcriptome data from TCGA. These lncRNAs exhibited substantial variation in number, expression patterns, and RCD associations among different cancers. We explored the translational potential of RCD-related lncRNAs. Prognostic risk models constructed using these lncRNAs stratified patients effectively, and NMF-based molecular subtyping revealed distinct immune microenvironments. Furthermore, integration with drug sensitivity data predicted potential therapeutic agents, suggesting that expression levels of specific RCD-related lncRNAs may guide treatment decisions. We systematically compared our database with existing relevant databases (LncPCD [77], ncFO [78], FerrDb [45], HAMdb [46]). The results showed that the existing databases have limited RCD types and lack systematic pan-cancer analysis (Table S7). In contrast, LncRCD is the database to integrate 12 RCD types covering 18 TCGA cancer types, filling the gap in the RCD–lncRNA field from a pan-cancer perspective compared with other databases. However, our research still has certain limitations.

Firstly, the regulatory relationships of RCD–lncRNAs identified in this study are mainly based on computational inference and statistical correlations derived from bulk transcriptome data. Although we included tumor purity as a covariate to optimize these associations, they do not necessarily prove direct biochemical interactions or causal relationships, which require further verification. In this study, tumor purity was treated as the primary confounder given its substantial impact on cellular composition and gene expression correlations in bulk RNA-seq data. Other clinical variables (e.g., age and tumor stage), due to heterogeneity and imbalance across cancer types, may introduce bias or reduce statistical power if jointly adjusted. Our framework integrates partial correlation, gene set enrichment analysis (lncRES), and cross-cancer consistency filtering to enhance robustness and mitigate potential confounding effects. Also, the current version of the database only covers 18 major cancer types from TCGA. Extending it to include a wider range of tumors and integrating other omics data are crucial for addressing the specific cell death patterns of cell types in the tumor microenvironment. Since RCD can occur in a highly cell type-specific manner, the bulk RNA-seq profiles represent the comprehensive signals of all cells, which may mask the cell origin of the identified lncRNA–RCD associations. Future research can integrate computational deconvolution methods to estimate the proportions of cell types and separate the signals of specific cell components. At the same time, by combining single-cell transcriptomics or spatial transcriptomics data, it will be possible to more precisely analyze the cell type-specific regulatory patterns. These strategies will help to more accurately interpret the biological significance of lncRNAs related to RCD and improve the resolution of the inferred regulatory maps.

Secondly, some of the correlations we predicted between lncRNAs and RCD have been confirmed in other studies. For example, the expression of lncRNAs such as AC002331.1 and CTA-384D8.35 was significantly associated with most genes related to pyroptosis [79]. LINC00162 has oncogenic effects, and inhibiting LINC00162 can induce apoptosis [80]. FAM83H-AS1 inhibited ferroptosis in endometrial cancer cells by interacting with DNMT1, thereby increasing the methylation level of the CDO1 promoter [81]. It should be emphasized that correlation-based identification does not establish causality. Experimental validation is required to confirm whether these lncRNAs directly regulate RCD pathways. However, we still believe that the lncRNA–RCD relationship discovered in this study should be understood as a preferred candidate association rather than a direct causal regulatory relationship. In the future, it will be necessary to conduct integrated interference experiments, single-cell transcriptome, or multi-omics data integration studies to clarify the causal mechanism.

CTRP2 is a widely used pharmacogenomic resource for drug response modeling; while models built on cell line transcriptomic and drug sensitivity data may provide translationally informative predictions in certain settings [82], such models cannot fully capture in vivo pharmacokinetics, the tumor microenvironment, or interpatient heterogeneity. Therefore, the CTRP-based drug–lncRNA association predictions in this study should be considered a practical approach for prioritizing candidate associations rather than direct evidence of clinical efficacy. Similarly, all predicted drug–lncRNA associations are intended to provide potential candidates for further investigation—both should be interpreted with caution. Their potential role in modulating drug response warrants further validation in patient-derived models (such as organoids and xenografts) and clinical cohorts [83,84].

Finally, the case study only focused on KIRC, which may limit the general applicability of the research results. Given the significant differences among different cancer types, the biological and clinical relevance of lncRNAs related to RCD in other tumors may be different. Future research will extend the same analytical framework to other cancer types to further verify its broader applicability. Another data limitation of case study lies in the fact that the validation was limited to the TCGA cohort. This is mainly because the currently available public datasets rarely simultaneously contain tumor lncRNA expression data and corresponding survival information. Therefore, further validation is necessary in an independent and well-defined cohort.

LncRCD is committed to long-term sustainability and continuous improvement. Given that the number of newly identified RCD-related genes has plateaued following the systematic elucidation of core RCD pathways, the current dataset already provides a relatively complete landscape for most research applications. The database will undergo major updates every 1 to 2 years, with minor updates and bug fixes performed on an ongoing basis. Each major update will include (a) newly discovered RCD-related genes and RCD types, (b) expansion to more cancer types or integration of expression data from other public resources (e.g., ICGC and GTEx), and (c) incorporation of new omics data types, such as single-cell transcriptomics.

In conclusion, this study provides a comprehensive atlas of RCD-associated lncRNAs across multiple cancers, elucidating their potential regulatory roles and clinical relevance. By integrating computational predictions with existing experimental evidence, our work lays the foundation for further mechanistic studies and the development of lncRNA-based prognostic and therapeutic strategies.

## Data Availability

The web-based database developed in this study can be accessed for free at https://lncrcddb.bio-database.com. To ensure the complete reproducibility of our computational workflow, the code used for data preprocessing and analysis has been stored in an open GitHub repository, and the link to this repository is located at https://github.com/wangliTeam/LncRCD.git.

## Supplementary Materials

Supplementary 1 Supplementary Method Fig. S1 Tables S1 to S7
