# Supplementary material for: LncRCD: A Comprehensive Database for Pan-Cancer Characterization of lncRNAs Related to 12 Regulated Cell Death Types
Source: Comput Struct Biotechnol J. 2026 Jun 1;35(1):0110. doi: 10.34133/csbj.0110 (PMC13223398; doi:10.34133/csbj.0110)
Supplement: Supplementary 1 — Supplementary Method Fig. S1 Tables S1 to S7 [file csbj.0110.f1.zip › Supplementary material.docx]

**Supplementary material**

**Supplementary Method**

**Identify RCD-related lncRNAs**

For each differentially expressed lncRNA, we ranked all genes according to the correlation of their expression with that lncRNA. the expression of lncRNA i and gene j in tumor patients was defined as L(i) = (l_1_, l_2_, l_3_, ..., l_i_, ..., l_m_) and G(j) = (g_1_, g_2_, g_3_, ..., g_j_, ..., g_m_). Tumor purity scores of m patients were defined as P = (p_1_, p_2_, p_3_, ..., p_i_, ..., p_m_). We first calculated the partial correlation coefficient (PCC) between lncRNA i and gene j expression using tumor purity as a covariate,

 ①

Where R_LG_, R_LP_, and R_GP_ are the correlation coefficients between lncRNA i expression and coding gene j, lncRNA i expression and tumor purity, and gene j expression and tumor purity, respectively. In addition, we obtained the P value of PCC, defined as P(ij). For each lncRNA-gene pair, we calculated the rank score (RS) as follows,

 ②

Subsequently, we mapped the genes in each regulatory cell death type to the sorted gene list. Next, we calculated the enrichment score (ES) according to GSEA. If there are N genes in the sorted gene list L = {g_1_, g_2_, g_3_, ..., g_N_}, then the sorting score is RS(g_j_) = r_j_. We first evaluated the proportion of genes in pathway H (“hits”) (weighted by RS) and not in pathway S (“missing”) the proportion of genes in path S (up to a given position i in L) as follows,

③

ES scores were P_hit_ - P_miss_ with maximum deviation from zero. In addition, p-values were calculated for each pathway containing N_I_ genes as follows,

④

Where ES_ik_ is the ES score between lncRNA i and the RCD type k, N is the number of genes in the ranking list, and N_I_ is the number of genes in the regulatory cell death type. P values were adjusted using FDR. In addition, based on previous studies, we combined the P values and ES scores into the lncRES score,

⑤

Therefore, the lncRES scores ranged from -1 to 1.

**Supplementary figures**


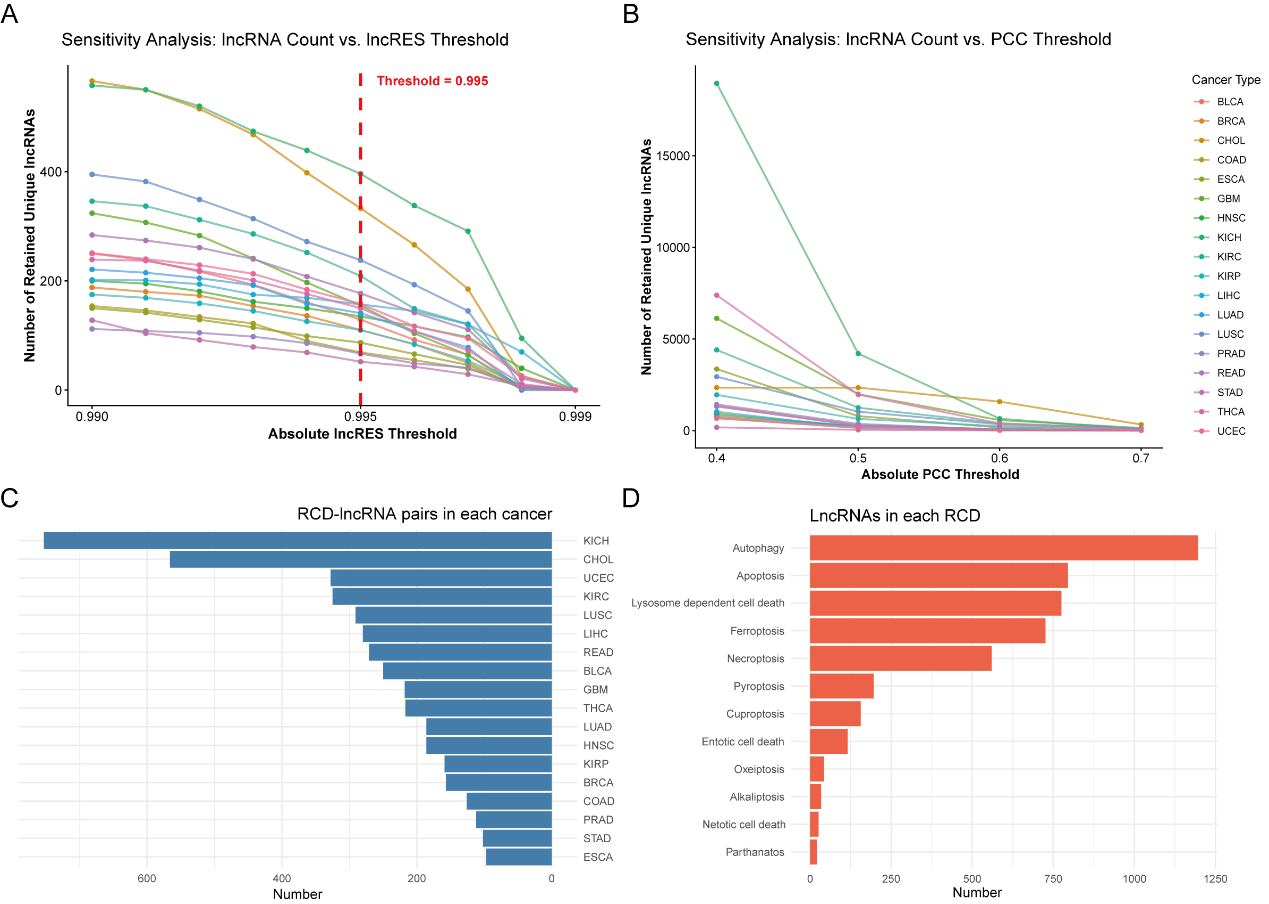


**Supplementary figure 1. Sensitivity analysis of the threshold and landscape of RCD–lncRNA associations across cancers and cell-death programs.** **(A)** Number of retained unique lncRNAs in each cancer type under varying absolute lncRES thresholds. A progressive reduction in retained lncRNAs was observed with increasing stringency of the lncRES cutoff. The red dashed line marks the selected threshold applied in this study (|lncRES| = 0.995). **(B)** Number of retained unique lncRNAs in each cancer type under different absolute Pearson correlation coefficient (PCC) thresholds. The number of the candidate lncRNAs decreased substantially as the PCC cutoff increased, reflecting the impact of correlation stringency on candidate selection. Each line represents one cancer type. **(C)** Number of RCD–lncRNA pairs identified in each cancer type (TCGA abbreviations shown: ESCA, STAD, PRAD, COAD, BRCA, KIRP, HNSC, LUAD, THCA, GBM, BLCA, READ, LIHC, LUSC, KIRC, UCEC, CHOL, KICH). **(D)** Number of lncRNAs associated with each RCD types (Autophagy, Apoptosis, Lysosome-dependent cell death, Ferroptosis, Necroptosis, Pyroptosis, Cuproptosis, Entotic cell death, Oxeiptosis, Alkaliptosis, Netotic cell death, Parthanatos).
